# Identifying Cancer Driver Pathways Based on the Mouth Brooding Fish Algorithm

**DOI:** 10.3390/e25060841

**Published:** 2023-05-24

**Authors:** Wei Zhang, Xiaowen Xiang, Bihai Zhao, Jianlin Huang, Lan Yang, Yifu Zeng

**Affiliations:** 1 College of Computer Science and Engineering, Changsha University, Changsha 410022, China; 2 Hunan Province Key Laboratory of Industrial Internet Technology and Security, Changsha University, Changsha 410022, China

**Keywords:** cancer progression, driver gene coverage, driver pathway, biological effects

## Abstract

Identifying the driver genes of cancer progression is of great significance in improving our understanding of the causes of cancer and promoting the development of personalized treatment. In this paper, we identify the driver genes at the pathway level via an existing intelligent optimization algorithm, named the Mouth Brooding Fish (MBF) algorithm. Many methods based on the maximum weight submatrix model to identify driver pathways attach equal importance to coverage and exclusivity and assign them equal weight, but those methods ignore the impact of mutational heterogeneity. Here, we use principal component analysis (PCA) to incorporate covariate data to reduce the complexity of the algorithm and construct a maximum weight submatrix model considering different weights of coverage and exclusivity. Using this strategy, the unfavorable effect of mutational heterogeneity is overcome to some extent. Data involving lung adenocarcinoma and glioblastoma multiforme were tested with this method and the results compared with the MDPFinder, Dendrix, and Mutex methods. When the driver pathway size was 10, the recognition accuracy of the MBF method reached 80% in both datasets, and the weight values of the submatrix were 1.7 and 1.89, respectively, which are better than those of the compared methods. At the same time, in the signal pathway enrichment analysis, the important role of the driver genes identified by our MBF method in the cancer signaling pathway is revealed, and the validity of these driver genes is demonstrated from the perspective of their biological effects.

## 1. Introduction

Inside the cell, various forms of signals are interconnected and interact with each other, forming different signaling pathways for a variety of biological functions. When a gene responsible for regulating these functions is mutated, this leads to biological dysfunction and can even induce carcinogenesis [1,2]. Generally, the set of mutated genes that plays a major role in cell signaling pathways is called the driver pathway or driver gene set. The identification of driver pathways can not only further increase our understanding of the mechanisms of cancer formation and laws of molecular action but may also provide new molecular targets for cancer treatment. It is well known that different genetic mutations may target the same pathways. Therefore, it is necessary to move from the gene level to the pathway level, which can help to capture the heterogeneous patterns of tumors. Some studies have found patterns of mutations at the pathway level [3,4]. Most of these studies are based on known information about signaling pathways, and attempt to search pathway databases for pathways that drive cancer development. However, this approach can only look for known pathways [5,6]. Given the incompleteness of existing pathway information, it has become increasingly important to develop new algorithms to discover mutated driver pathways or gene sets that do not rely on prior knowledge [2,7].

In previous work, two main types of methods have been proposed for the detection of driver pathways: de novo identification methods and knowledge-based methods. De novo methods identify cancer-driver pathways without any prior knowledge, taking advantage of two characteristics of the pathway: high coverage and high mutual exclusivity. High coverage means that the driver gene set covers a large number of samples. High mutual exclusivity means that a driver mutation involved in a pathway is sufficient to interfere with that pathway. Most de novo methods formalize the problem of driver pathway identification into a maximum coverage exclusive submatrix model. For example, Dendrix [8] detects the combination of factors with high coverage and mutual exclusion by solving the maximum coverage submatrix problem [9,10,11]. Mutex [12], MDPFinder [13], multi-Dendrix [14], BeWith [15], ComMDP, and SpeMDP [16] solve the maximum coverage exclusive submatrix problem using integer linear programming to identify mutually exclusive gene sets.

In contrast, knowledge-based approaches use genomic data and combine prior knowledge in the form of pathways, networks, and functional phenotypes to identify driver pathways. In the Hotnet [17], Hotnet2 [18], and Hierarchical Hotnet [19] methods, thermal diffusion is a common property, and diffusion values are used to extract modules with high connectivity; these pathways are defined by graph theory connectivity (usually a strong connectivity). MEMO [11] uses the interaction network and function diagram to derive the largest group in the similar graph, and the maximum group is processed with mutual exclusivity. Babur et al. proposed a web-based method for seed growth that identifies pan-cancer modules using TCGA data, which determines growth strategies based on appropriately defined mutually exclusive scores. However, problems remain in the identification of driver pathways using these existing methods. On the one hand, the existing de novo methods based on the maximum weight submatrix model attach equal importance to coverage and exclusivity and assign them equal weight, but these methods ignore the impact of mutational heterogeneity. On the other hand, human interaction data are incomplete, resulting in knowledge-based approaches being limited in the discovery of new driver pathways.

In the current project, we investigate the problem of discovering driver pathways directly from cancer mutation and gene covariant data without prior knowledge of the pathways or other interactions between genes, and propose a method to identify driver genes at the pathway level based on the Mouth Brooding Fish (MBF) algorithm [20]. This algorithm is a relatively new optimization algorithm [20] that simulates the symbiotic interaction strategies employed by organisms to survive and reproduce in ecosystems, using the locomotion, dispersion, and protective behavior of mouth brooding fish as a model to find the best possible answer. The existing research [20] shows that it has clear advantages in global optimization compared with other advanced intelligent optimization algorithms. The MBF algorithm is used to process the maximum weight submatrix model to find as many driver pathways with high coverage and high exclusivity as possible. Specifically, our method reduces the complexity of discovering driver pathways by incorporating gene covariate data through principal component analysist and constructs a maximum weight submatrix model considering different weights of coverage and exclusivity. To some extent, the effect of mutational heterogeneity is thus overcome. The accuracy, coverage, mutual exclusion, and other indexes show the effectiveness of the method in identifying driver pathways. The analysis of time cost shows the high efficiency of our method (for the convenience of subsequent description, it will be shortened to “MBF-based”). Through the enrichment analysis of signal pathways, the molecular characteristics and biological roles of the identified driver genes can be determined from the perspective of biological action.

The main contributions of this paper are summarized as follows: (1) We propose an MBF-based method to solve the maximum weight submatrix model with fusion covariates. It considers different weights of coverage and exclusivity and overcomes the effect of mutational heterogeneity. (2) With the obvious advantages in global optimization, the MBF algorithm can be used to identify cancer driver pathways.

The rest of this paper is organized as follows: Section 2 provides the materials and methods. Section 3 presents the experimental results, including analysis and comparative study. Section 4 provides a discussion of this project, including future directions to extend this work, and Section 5 presents our conclusions.

## 2. Materials and Methods

### 2.1. Maximum Weight Submatrix Model

For mutation data containing m patients and involving n genes, the mutation data can be converted into an *m* × *n* mutation matrix A, where each row represents the mutation of different genes in each patient, and each column represents the mutation of the corresponding gene in different patients. $A_{i,j}=1$ means that gene j of patient i is mutated, and $A_{i,j}=0$ means that gene j of patient i is not mutated.

For gene g, let $\Gamma \left(g\right)=\left\{i:A_{i,g}=1\right\}\;$denote the set of patients with mutations in gene g, and similarly for gene set M, let $\Gamma \left(M\right)={⋃}_{g\in M}\Gamma \left(g\right)$ denote the set of patients with mutations in at least one gene in *M*. A larger Γ(M) value indicates that M has higher coverage. M is said to be highly exclusive when the proportion of people who have only one mutation in M, and not two or more mutations, increases. Similarly, for an m×k submatrix M of A, high coverage is defined as having at least one mutation in each row, and high exclusivity is defined as having as many mutations in each row as possible. Equation (1) defines the weight of M, which is used to balance coverage and exclusion, to find as many driver pathways as possible with coverage and exclusion.

(1) $$W\left(M\right)=\left|\Gamma \left(M\right)\right|-\omega \left(M\right)=2\left|\Gamma \left(M\right)\right|-\sum_{j\in M}\left|\Gamma \left(j\;\right)\right|$$

where M is the m×k submatrix of A, W(M) is the weight of M, M is the weight submatrix, |Γ(M)| represents the coverage of M, and $\omega \left(M\right)=\sum_{j\in M}\left|\Gamma \left(j\;\right)\right|-\left|\Gamma \left(M\right)\right|$ represents the exclusivity of M. The problem of maximizing Equation (1) is also known as the maximum weight submatrix problem [13,14,21,22,23,24].

### 2.2. Correlation Analysis between Covariates and Mutation Rate

Mutational heterogeneity greatly affects the effectiveness of various methods of driver gene identification and becomes a key problem to be solved when screening driver genes from passenger genes. Lawrence et al. [25] analyzed three types of mutational heterogeneity, the first being differences in gene mutation rates among different forms of cancer. The mutation rates of patients with 27 different types of cancer were calculated and found to vary by up to 1000-fold. The second type of mutational heterogeneity is the difference in the mutational spectrum of different cancers. For example, in lung cancer, C→A mutations occupy the majority of the mutation spectrum, while in melanoma, C→T forms the majority of the mutation spectrum. The third type of mutational heterogeneity is regional heterogeneity [26]. Regional heterogeneity refers to the difference in mutation rates of different regions within the genome of tumor cells, that is, the difference in mutation rates between different genes. In general, it mainly targets the same cancer identification pathway, so our current project analyzes regional heterogeneity in mutational heterogeneity, expecting to find a solution. The covariates contributing to regional heterogeneity have been reported to include gene expression level, gene replication time, and chromosome status [15]. Xi et al. [27] analyzed the correlation between these covariates and the mutation rate, which not only demonstrated a strong correlation between these three covariates and the mutation rate, but also proved that there was a strong correlation between these three covariates [28,29,30]. Therefore, in this paper, principal component analysis was performed to determine the weight of gene expression level, gene replication time, and chromosome status, and the three covariates were combined into a single covariate for analysis to reduce the complexity of the algorithm.

### 2.3. Identifying Driver Pathways Based on the Mouth Brooding Fish Algorithm

**Figure 1:**
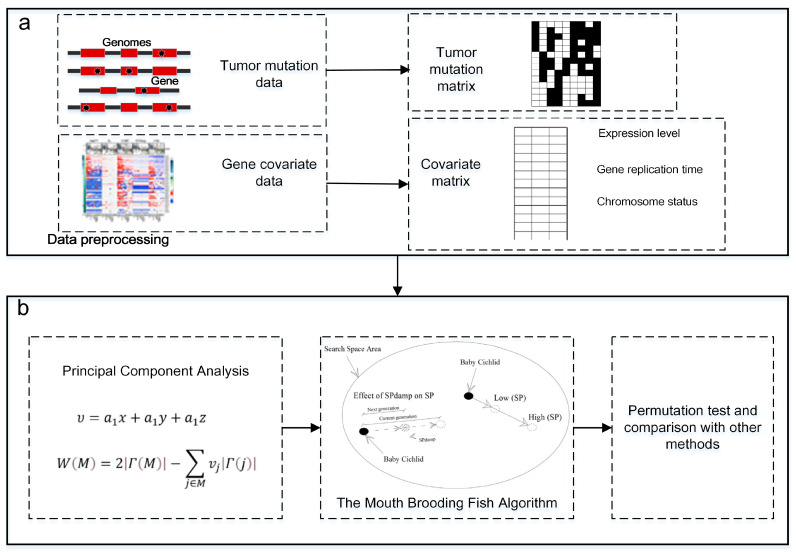
Overview of methods, with the data processing and main strategy listed. (**a**) Data preprocessing steps. Step 1: Tumor mutation data generates the 0–1 mutation matrix. Step 2: Gene covariate data are converted to triples (expression level, gene replication time, chromosome status). (**b**) Main strategy of this paper. Step 1: Construction of maximum weight submatrix model with fusion covariates. Step 2: Solving of the Mouth Brooding Fish algorithm. Step 3: Permutation test and comparison with other methods.

The flow diagram of our MBF-based method is shown in Figure 1. We use the Mouth Brooding Fish algorithm [20] as the solution algorithm for the model in this paper. Our method first processes the input data, including tumor mutation data and gene covariate data. Gene covariate data include gene expression level, gene replication time, and chromosome status data. Tumor mutation data are converted into *m* × *n* mutations in the matrix, and the covariate data are converted into the corresponding n×3 covariant matrix; then, through principal component analysis, the n×3 covariant matrix is converted into the n×1 covariant matrix, which is integrated into the maximum submatrix weight model to build a new optimization model, which is then solved by the Mouth Brooding Fish algorithm. In the last part of the experimental analysis, the enrichment analysis of the identified pathways and the time cost of the algorithm are compared with alternative analysis methods.

#### 2.3.1. Principal Component Analysis of Covariates

According to the previous analysis, we performed principal component analysis on gene expression level [31], gene replication time, and chromosome status to determine their respective weights, and finally combined them into a covariate. The principal component analysis steps are as follows:

Step 1: Normalize the covariate to obtain the normalized gene expression level x, gene replication time y, and chromosome status z.

Step 2: Calculate the covariance matrix A of the three covariates after normalization.

Step 3: Calculate the maximum characteristic root λ of A.

Step 4: Calculate the unit feature vector $a=\left[a_{1}\;a_{2}\;a_{3}\right]$ corresponding to λ.

Step 5: Then, the expression of the new fusion covariate υ is as follows:

(2) $$\upsilon =a_{1}x+a_{2}y+a_{3}z\;$$

#### 2.3.2. Construction of Maximum Weight Submatrix Model with Fusion Covariates

Dendrix [8] constructs the maximum weight submatrix model of Equation (3); however, this model gives the same weight to coverage and exclusivity and does not consider the problem of gene mutational heterogeneity. Therefore, three major factors contributing to mutational heterogeneity were included in this paper: gene expression level, gene replication time, and chromosome status. Considering the strong correlations among the three [32], these are fused into one covariate by Equation (4), and a new fusion covariate υ is generated. Thus, the following maximum weight submatrix model with fusion covariates is obtained:

(3) $$W\left(M\right)=\left|\Gamma \left(M\right)\right|-\omega \left(M\right)=2\left|\Gamma \left(M\right)\right|-\sum_{j\in M}v_{j}\left|\Gamma \left(j\right)\right|$$

The above equation can be transformed into a unary linear programming model:

$$maxf\left(y\right)=2\sum_{i=1}^{m}x_{i}-\sum_{j=1}^{n}(y_{j}\sum_{i=1}^{m}\upsilon_{j}A_{i,j})$$

(4) $$s.t.\{\begin{array}{c} \sum_{j=1}^{n}y_{j}=k\\ \;\;\;\;\;\;\;\sum_{j=1}^{n}y_{j}A_{i,j}\geq x_{i}\\ \;\;\;\;\;x_{i},y_{j}\in \left\{0,1\right\} \end{array}$$

where k represents the number of genes in the driver pathway; *M* denotes the size of the matrix columns; *x_i_* indicates whether patient i has a gene mutation in M; $y_{j}$ indicates whether gene j is mutated in M, where $y_{j}=1$ means gene *j* is mutated in M, and $y_{j}=0$ otherwise; and $\upsilon_{j}\;$is the fusion covariate corresponding to gene j, which is calculated by Equation (4).

#### 2.3.3. Solving of the Mouth Brooding Fish Algorithm

We use the MBF algorithm to process the maximum weight submatrix model provided in Section 2.3.2.

(1) Search Space and Code of the Mouth Brooding Fish Algorithm

The decision variable is the variable in the n-dimensional vector y that can assume two values: 0, which means that the final pathway does not contain genes at this position, or 1, which means that the final pathway contains genes at this position. The search space is determined by condition $\sum_{j=1}^{n}y_{j}=k$ and the total number of genes n, as shown in Equation (5):

(5) $$\left\{\left(y_{1},y_{2},\ldots ,y_{n}\right)|y_{j}\in \left\{0,1\right\},j=1,2,\ldots n,\sum_{j=1}^{n}y_{j}=k\right\}$$

where *y_j_* represents the decision variable of the gene corresponding to column j of mutation matrix A, and k is the total number of genes in the pathway.

(2) Fitness Function of the Mouth Brooding Fish Algorithm

To determine the importance or target value of each mouth brooding fish in the search space, we use Equation (4) as the fitness function to find a suitable mouth brooding fish in the search space. The Mouth Brooding Fish algorithm finds a mouth brooding fish that maximizes its fitness function in the search space, which is the optimal solution. According to the optimal solution and the code setting of the mouth brooding fish, it can be used to obtain the final driver pathway gene sets.

(3) Setting of Parameters for the Mouth Brooding Fish Algorithm

The Mouth Brooding Fish algorithm simulates real behavior. It has 5 parameters: SP is the position of the mother,nFish is the number of mouth brooding fish, *Dis* is the dispersal distance from the mother, *Pdis* is the dispersal probability of the fish from the mother, and SPdamp is the position damping of the mother. In our analysis, SP is 0.6, nFish is 50, Dis is 1.8, Pdis is 0.2, and SPdamp is 0.95. These values are statistically derived from real mouth brooding fish [20].

Applying these parameters to the MBF algorithm, and conducting the steps described below, the best solution involves identifying the mouth brooding fish with the best position.

The Algorithm 1 is our novel algorithm to identify driver genes at the pathway level.
**Algorithm 1:** Identifying the cancer driver pathway based on the Mouth Brooding Fish (MBF) algorithm.Input: The mutation matrix *A*, the gene expression level vector *x*, gene replication time vector *y*, and chromosome state vector *z*.Output: Optimal driver pathways (that is, a set of driver genes).1. *SP* is 0.6, *nFish* is 50, *Dis* is 1.8, *Pdis* is 0.2, and *SPdamp* is 0.95.2. Process *x*,*y*,*z* to obtain *x*′,*y*′,*z*′.3. Analyze principal components and calculate the fusional $v=a_{1}x^{\prime }+a_{1}y^{\prime }+a_{1}z^{\prime }$4. For k=1,2,3,4,…,n:Initialize the mouth brooding fish population randomly $\left\{\left(y_{1},y_{1},\ldots ,y_{n}\right)|y_{j}\in \left\{0,1\right\},j=1,2,\ldots ,n,\sum_{j=1}^{n}y_{j}=k\right\}$.Perform the classic MBF algorithm.Add the variable with the highest fitness in the MBF algorithm to a set of driver genes.5. Return a set of driver genes.

#### 2.3.4. Permutation Test

The significance of the driving pathway needs to be tested. In this paper, the permutation test is used, and the significance level is 0.05. If the p value of the pathways does not reach the significance level, the pathways with the next lowest fitness values are tested in turn, and the corresponding driver pathways are used when the significance level is reached.

### 2.4. Datasets

- Lung Adenocarcinoma Dataset

Lung adenocarcinoma is a subtype of non-small cell lung cancer (NSCLC). Lung adenocarcinoma cancer cells are classified according to their phenotype under the microscope. Lung adenocarcinoma begins with glandular cells that secrete mucus and other substances and tends to metastasize around smaller airways (such as alveoli). Lung adenocarcinoma is usually located at the outer edge of the lung.

The lung adenocarcinoma data used in this paper were sourced from the research of Vandin et al., involving 188 patients with lung adenocarcinoma and 623 genes.

b. Glioblastoma Multiforme Dataset

Glioblastoma multiforme (GBM) is one of the most aggressive types of cancers. Its indicators and symptoms are nonspecific, and these symptoms often worsen rapidly and may lead to coma. The etiology of most malignant gliomas is unknown. The common causes include genetic diseases, such as neurofibromatosis and Li–Fraumeni syndrome, and previous radiotherapy [33]. GBM accounts for 15% of all brain tumors [34].

The glioblastoma multiforme data were sourced from The Cancer Genome Atlas (TCGA) and includes copy number variation, small indels, and single-nucleotide variants (SNVs). This dataset contains 261 patients and 487 genes in total.

c. Gene Covariate Data

Gene covariate data were sourced from The Cancer Genome Atlas (TCGA) and includes gene expression, gene replication time, and chromosome status. The expression covariate of lung adenocarcinoma data was used to obtain the expression covariate matrix set of 623 × 3 (623 genes × triples (expression level, gene replication time, chromosome status)), while that of the glioblastoma multiforme dataset is 487 × 3 (487 genes × triples (expression level, gene replication time, chromosome status)).

d. Simulated Data

In order to evaluate the computational performance of this method, random methods are used to generate the mutation matrix at n=1000, 5000, 10,000 and m=100, 200,… ,1000, where *m* denotes patient and n denotes gene. Additionally, the genes in the simulated data are of no particular significance, and the covariate of the combined principal components of different genes is set to 1.

### 2.5. Experimental Setup and Evaluation Index

In order to compare our method with the Dendrix [8], MDPFinder [13], and Mutex [12] methods, we set five indexes: (1) submatrix weight value, (2) accuracy, (3) coverage, (4) mutual exclusion, and (5) q value of the enrichment pathway. The submatrix weight value is calculated according to Equation (1), which represents a comprehensive index of coverage and exclusivity. The higher the score is, the greater the weight value of this pathway is, and the more likely it is to be a driver pathway; the accuracy is a measure of how many genes are drivers in the identified pathways, that is, how many genes are in the driver pathways of related cancers. The accuracy is calculated through some cancer-related pathways in the KEGG database; the coverage represents the proportion of gene mutations in the pathway of the population; and the coverage is calculated using Equation (6). The mutex degree refers to the proportion of the sample with only one gene mutation in the pathway; the enrichment pathway q value shows the enrichment degree of this pathway with the existing related cancer database pathways. We used the investigated gene sets in the MSigDB database (https://www.gsea-msigdb.org/gsea/msigdb/annotate.JSP (accessed on 12 March 2022)) to compute the following function:

(6) $$cover=\frac{1}{m}\left|\Gamma \left(M\right)\right|$$

## 3. Results

In this section, we present the experimental results, including performance comparisons with other methods.

### 3.1. Lung Adenocarcinoma Test Results

In general, an average of 3~10 driver gene mutations lead to cancer [35]. Thus, to consider mutual exclusion, we conducted experiments on the data when the number of genes k=3~10  in the pathway and obtained the driver pathway under different values of *k*, the results are list in Table 1. All pathways were assessed for significance by the permutation test, and *p* values were all less than 0.05. When k=7, the identified drivers were EGFR, KRAS, NF1, STK11, LRP1B, ERBB4, and CDKN2A. When k=10, the identified drivers were EGFR, KRAS, NF1, STK11, ATM, TP53, APC, LRP1B, ERBB4, and AKT1.

**Figure 2:**
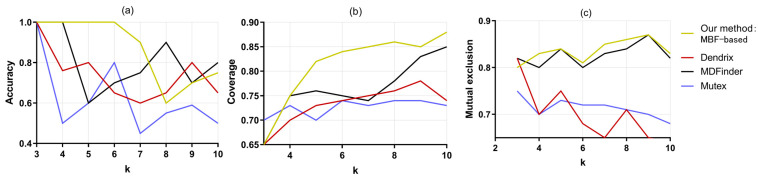
Comparison of three indicators of the four methods to identify driver gene paths in lung. (**a**) The four methods obtain different accuracies of the pathways for different k values. (**b**) The four methods obtain different coverage of the pathways for different k values. (**c**) The four methods obtain different mutual exclusion of the pathways for different k values.

Additionally, we compared our MBF-based algorithm with Dendrix [8], MDPFinder [13], and Mutex [12], and we show the results in Figure 2. It can be seen that when the pathway scale value k increases from 3 to 10, the accuracy of this MBF-based method and the MDPFinder, Dendrix, and Mutex methods shows a downward trend. In the identification of cancer driver genes, the presence of passenger genes is the main interference factor. With the gradual increase in k, the effect of this interference factor is increased. However, the accuracy of this MBF-based method is always above 75%, and the accuracy reaches 100% when k=3,4,5,6. In contrast, the accuracy of MDPFinder is only 100% when k=3,4, and only 60% when k=6. Additionally, the accuracy of Mutex is only 43% when k=7. Therefore, the accuracy of this MBF-based method is clearly better than that of the other compared methods. Figure 2b shows that the coverage of the four methods increases with the increase in k, because with the increase in the number of genes in the identified pathway, new samples are constantly joining the population covered by the pathway. In addition to the same coverage of MDPFinder when k=9, the MBF-based method is better than the other three methods in terms of other values. Figure 2c shows the comparison of the mutual exclusion of the four methods. With the gradual increase in k, the mutual exclusion between MBF-based and MDPFinder increases slightly, while Dendrix and Mutex show a downward trend. At the same time, the mutex degree of our method is higher than or equal to that of other methods for k=4,5,6,7,8,9. In general, the MBF-based algorithm achieves good results in terms of accuracy, coverage, and mutual exclusion, and is significantly better than the remaining three methods, indicating that the proposed method is effective for identifying driver paths in lung adenocarcinoma.

In this paper, we took the pathway size k=10 as an example, conducted experiments, calculate the evaluation index using lung adenocarcinoma data, and compared the MBF-based algorithm with the Dendrix [8], MDPFinder [13], and Mutex [12] methods. The results are shown in Table 2a,b, in which we list the discovered pathways, and compare the four methods in terms of five evaluation indexes: submatrix evaluation, accuracy, coverage, mutual exclusion, and enrichment pathway. The enrichment value q is calculated using the “investigate gene set” function in the MSigDB database (gsea-msigdb.org).

When k=10, the selected genes were EGFR, KRAS, NF1, STK11, ATM, TP53, APC, LRP1B, ERBB4, and AKT1, and the enriched pathways were non-small cell lung cancer and MAPK signaling pathway. At the same time, the weight, coverage, and mutual exclusion of the MBF-based submatrix were 1.700, 0.870, and 0.840, respectively, which are higher than the values associated with the other three methods. The accuracy of the MBF-based algorithm was 80%, like that of MDPFinder, which is also higher than the 60% accuracy of Dendrix and the 50% accuracy of Mutex.

In order to better introduce the interaction between genes in the pathways identified by the MBF-based strategy and to better understand the biological significance of the identified pathways, we used the Database for Annotation, Visualization and Integrated Discovery (DAVID) [36] to analyze them.

**Figure 3:**
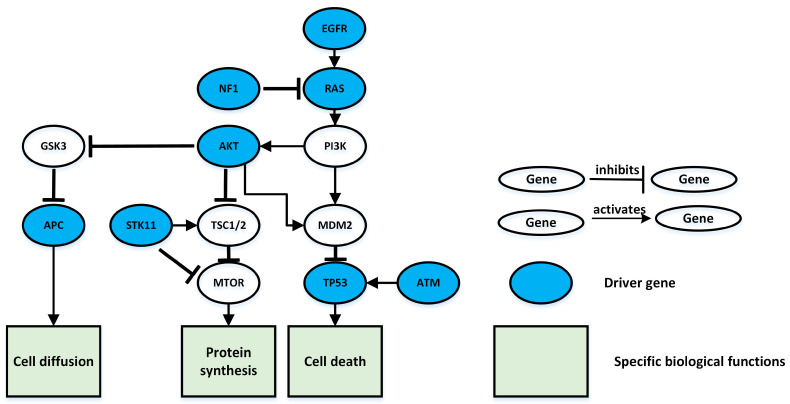
Signal pathways associated with lung adenocarcinoma. The blue nodes are the eight driver genes in the signaling pathway identified under k = 10. Rectangular nodes indicate a specific biological function, and the line between nodes indicates the interaction between them: the arrow indicates an activation relationship between the two nodes; the “T” connector line indicates an inhibitory relationship between the two nodes.

The eight genes identified here play a critical role in the formation of lung adenocarcinoma, we can see from Figure 3. For example, the EGFR gene encodes a receptor protein called the epidermal growth factor receptor, which spans the thin cell membrane, with one end inside the cell and one end outside the cell. Most somatic mutations of the EGFR gene associated with lung adenocarcinoma cause abnormal expression of the KRAS gene, which can continuously activate the EGFR signaling pathway and eventually enhance cell proliferation, leading to the generation of cancer. The ATM gene provides instructions for making proteins, mainly in the nucleus, where it helps control the rate at which cells grow and divide [37,38,39]. This protein also plays an important role in the normal development and activities of several body systems, including the nervous system and the immune system. ATM activates downstream TP53, and when DNA damage occurs, ATM sends a signal to the TP53 gene, which is responsible for repairing damaged DNA or killing cancer cells.

### 3.2. Glioblastoma Multiforme Test Results

Glioblastoma multiforme data were tested when the number of genes k=1~10 in the pathway, and driver pathways under different *k* were obtained (Table 3). All pathways passed the significance assessment of the permutation test, with p values all less than 0.05.

**Figure 4:**
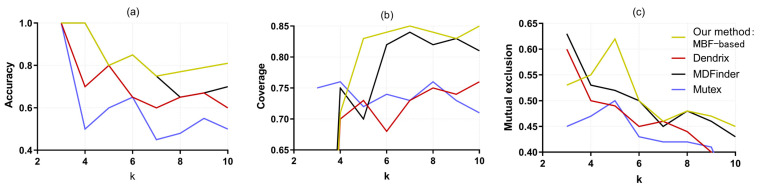
Comparison of three indicators of the four methods for the analysis of glioblastoma multiforme (GBM). (**a**) The four methods obtain different accuracies of the pathways under different k values. (**b**) The four methods obtain different coverage of the pathways under different k values. (**c**) The four methods obtain different mutual exclusion of the pathways under different k values.

To compare our MBF-based algorithm with the Dendrix [8], MDPFinder [13], and Mutex [12] methods, the comparison diagram is shown in Figure 4. Figure 4a presents plots of these comparisons. When the pathway size k is increased from 3 to 10, the accuracy of the proposed MBF-based method and the MDPFinder, Dendrix, and Mutex methods show a decreasing trend. It is well known that in the recognition of cancer driver genes, the presence of concomitant genes is the main interference factor [40]. With the increase in k, the influence of such interference factors also increases gradually, so the accuracy of these four methods shows a downward trend. However, the accuracy of the proposed method is always above 70%, and the accuracy reaches 100% when k=3,4. While the accuracy of Dendrix and Mutex was 100% at only *k* = 3, the accuracy of Mutex was only 50% at k=4, and was only 42% at k=7. Therefore, the proposed method is superior to the other methods in terms of accuracy. Figure 4b shows the comparison of coverage. With the increase in *k*, the coverage of the four methods increases, because with the increase in the number of genes in the identified pathway, new populations are constantly added to the coverage population of the pathway. When k≥ 4, the coverage of the proposed method is greater than or equal to that of the remaining three methods. Figure 4c shows the comparison of the mutual exclusivity of all four methods. The mutual exclusivity of the four methods decreases with the gradual increase in k, but the mutual exclusivity of the MBF-based method in this paper shows an increasing trend at the beginning and is higher than the other three methods under most values of k. In general, in terms of accuracy, coverage, and mutual exclusion, the MBF-based algorithm achieves good results and is significantly better than the other three methods, indicating that the MBF-based method is effective for recognizing driver pathways using glioblastoma multiforme data.

In this paper, the pathway size *k* = 10 was used as an example to analyze the glioblastoma multiforme data. The evaluation indexes were calculated and compared with those of the Dendrix [8], MDPFinder [13], and Mutex [12] methods. The results are shown in Table 4a,b.

At *k* = 10, the genes screened in this study were CDK4, CDKN2B, TP53, NF1, FGFRCDKN2A, TP53, PRF1, RB1, and SIGLEC9, and the enriched pathways were Glioma and Cell Cycle. Meanwhile, the submatrix weight, coverage, mutual exclusion, and accuracy for our MBF-based algorithm were 1.890, 0.850, 0.450, and 0.800, respectively, which are higher than the values of the other three methods.

**Figure 5:**
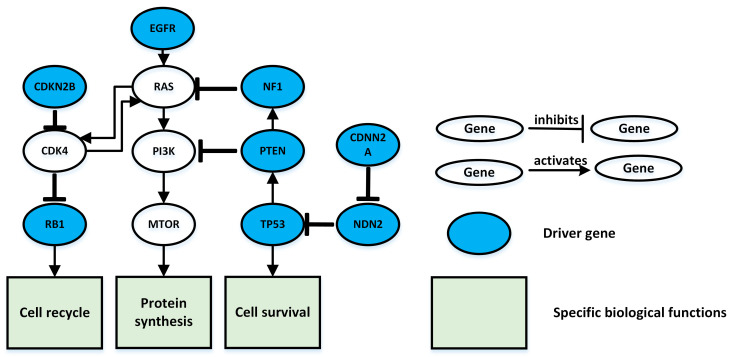
Glioblastoma multiforme (GBM)-related signal pathways. The blue nodes are the eight driver genes in the signaling pathway identified at k = 10. Rectangular nodes indicate a specific biological function, and the line between nodes indicates the interaction between them: the arrow indicates an activation relationship between the two nodes; the “T” connector line indicates an inhibitory relationship between the two nodes.

In order to analyze the interactions among genes in the pathways identified by the MBF-based method in this paper and to understand the biological significance of the identified pathways, these pathways were analyzed using The Database for Annotation, Visualization and Integrated Discovery (DAVID) (see Figure 5).

The driver genes that we identified play an important functional role in glioblastoma multiforme (GBM). For example, the NF1 gene encodes the protein neurofibrillin. This protein is produced in many types of cells, including nerve cells and specialized cells called oligodendrocytes and Schwann cells, which surround nerves. These specialized cells form the myelin sheath, a fatty layer that acts to insulate and protect certain nerve cells. Another example is CDKN2B and CDK4 genes, which play a direct inhibitory role in the RB signaling pathway. The CDKN2B gene is adjacent to the tumor suppressor gene CDKN2A and encodes a kinase inhibitor that blocks CDK kinase activation and inhibits cell cycle G1 progression.

### 3.3. Simulation Experiment Results

In order to evaluate the computational performance of the proposed method, a mutation matrix at m = 100, 200, ⋯,1000 and n = 1000, 5000 was randomly generated. At the same time, the covariate of the principal components of different genes was set to 1.

**Figure 6:**
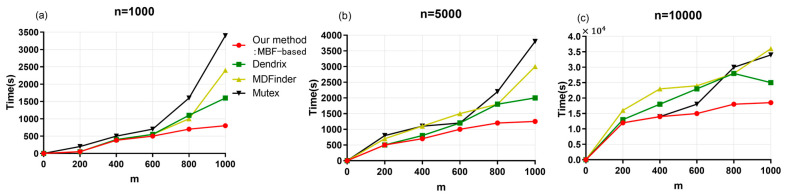
Comparison of execution times of the four compared methods for different numbers of samples. (**a**) For total gene number = 1000. (**b**) For total gene number = 5000. (**c**) For total gene number = 10,000.

The simulation data were analyzed using the MBF-based method proposed in this paper, as well as with Dendrix, MDPFinder, and Mutex, and the time required for each of them yielded the results shown in Figure 6.

As can be seen from Figure 6, for the total number of genes n = 1000, the time cost of the four methods gradually increases with the increase in the number of patients m, but the time cost of our proposed MBF-based method increases more slowly than that of the other methods. When m > 500, the time cost of our MBF-based method is always lower than the time cost of the other three methods. The time cost of the Dendrix method is lower than that of MDPFinder, and the time cost of MDPFinder is lower than that of Mutex. With the increase in n, the difference in time cost among the four methods also gradually increases. Among them, the time cost of the Mutex method increases faster, and the time performance is poor. Under n = 5000 and n = 10,000, the time cost of the proposed method increases less quickly than that of the other three methods, and the increase is gradual. In conclusion, the proposed MBF-based method shows good time performance, which is due to the superior performance of the MBF algorithm in solving the model.

## 4. Discussion

In our Mouth Brooding Fish algorithm-based method, three areas of analysis warrant additional consideration and future development. First, in Section 2.3.1, the MBF-based method uses principal component analysis (PCA) to reduce the data dimensionality and to combine covariables, which transforms a set of possibly correlated variables into a set of linearly uncorrelated variables by orthogonal transformation. It extracts the features that can best represent the original data. According to information theory and its principles of entropy analysis, the more discrete the data is, the more information it carries. Principal component analysis (PCA) involves variable transformation, which is a linear combination of original variables to represent the new comprehensive variable. It emphasizes the contribution of variance to the new variable, regardless of whether the new variable has a clear practical meaning. In future studies, we will try to reduce the dimension of data from another angle to expand the research so that the processed data can have practical significance and independent attributes can be obtained that will explain the internal structure of the original variable. Independent component analysis is a novel analysis method based on information theory [41]. It is similar to principal component analysis (PCA) in form, and it can be tested as a supplemental or replacement method that is theoretically more suitable to find the hidden factors behind the observed data [42].

Second, *k* is used to represent gene number in the pathway, and the value of *k* needs to be preset; in the experiments of this paper, *k* has preset values ranging from 3 to 10. However, is it possible to discover the real driver pathways without setting *k* in advance? In the future, one could consider combining multiple omics data types, such as transcriptome, genome, and proteome, to identify driver pathways or modules by unsupervised methods such as graph clustering from the perspective of data analysis. Based on multiple data types, it is easy to discover the real pathway, and unsupervised methods can be considered without presetting *k*.

The third topic of analysis for future consideration involves the mutual exclusivity hypothesis existing in the gene sets of the same driver pathway. In further studies, it is necessary to explore the driver pathways of co-mutation. The main problem is that the pathways involved in tumorigenesis are complex, have unclear boundaries, and are interrelated, so it is necessary to explore a reasonable evaluation pathway method in depth. This will facilitate deciphering the pathogenesis of cancer at the genetic level, help to understand the molecular mechanisms of cancer occurrence, and advance the development of effective personalized treatment for cancer patients.

## 5. Conclusions

In this study, we described the required steps and experimental results of identifying cancer driver pathways based on the Mouth Brooding Fish (MBF) algorithm. We introduced the process of the maximum weighted submatrix model and demonstrated a clear understanding of the origin and difficulties of the identification of pathways. We analyzed the correlation between the covariate variables and mutation rate. Through this analysis, we determined that there is a strong correlation among gene expression level, replication time, chromosome state, and gene mutation rate. Based on this association, the gene expression level was incorporated into the identification of the driving factors, and the effect of mutational heterogeneity could be eliminated to a certain extent. We conducted experimental analysis using lung adenocarcinoma data, glioblastoma multiforme (GBM) data, and gene covariate data. Experiments were carried out on each dataset to identify the corresponding driver pathway, and we compared our MBF-based method with other similar methods; then, the biological role of the identified pathway in the related signaling pathway was analyzed. Finally, the time performance of the proposed MBF-based method was analyzed, which showed that the proposed method is efficient for driver gene identification at the pathway level. Our Mouth Brooding Fish algorithm-based approach is generalizable and can be applied to other gene sets and to other cancer types.

## Figures and Tables

**Table 1:** The identification of driver pathways in lung adenocarcinoma.

k (Number of Genes in Pathway)	The Driver Pathway
3	EGFR KRAS TP53
4	EGFR KRAS NF1 STK11
5	EGFR KRAS NF1 STK11 LRP1B
6	EGFR KRAS NF1 STK11 LRP1B ERBB4
7	EGFR KRAS NF1 STK11 LRP1B ERBB4 CDKN2A
8	EGFR KRAS NF1 STK11 LRP1B ERBB4 CDKN2A TERT
9	EGFR KRAS NF1 STK11 LRP1B ERBB4 CDKN2A TERT CYSLTR2
10	EGFR KRAS NF1 STK11 ATM TP53 APC LRP1B ERBB4 AKT1

Note: The genes in each row are sorted in descending order by their frequency of occurrence in the results.

**Table 2:** Comparison of the four methods for identifying pathways, submatrix weight, and accuracy for lung adenocarcinoma data (**a**). Comparison of the four methods in terms of coverage, mutual exclusion and enrichment pathway for lung adenocarcinoma data (**b**).

(**a**)				
**Method**	**Pathway**		**Submatrix Weight**	**Accuracy**
Our method: MBF-based	EGFR KRAS NF1 STK11 ATM TP53 APC LRP1B ERBB4, AKT1 PAK6 ABL1 CYSLTR2 EGFR		1.7	0.8
Dendrix [8]	SRC MAP3K15 MAST1 STK11 WT1 YES1 STK11 EGFR KRAS MKNK2		1.436	0.6
MDPFinder [13]	FES KRAS NF1 MAP3K3 STK11 TFDP1 KRAS EGFR BUB1 MAP3K3		1.682	0.8
Mutex [12]	MAP3K3 NF1 ERBB4 MAST1 ABL1 PAK6		1.393	0.5
(**b**)				
**Method**	**Coverage**	**Mutex**	**Enrichment Pathway (q Value)**	
Our method: MBF-based	0.870	0.840	Non-small cell Lung Cancer (8.58 × 10^−8^) MAPK Signaling Pathway (8 × 10^−8^)	
Dendrix [8]	0.740	0.620	GnRH Signaling Pathway (1.43 × 10^−6^) Adherens Junction (5.94 × 10^−5^)	
MDPFinder [13]	0.850	0.830	Neurotrophin Signaling Pathway (1.1 × 10^−4^) ErbB Signaling Pathway (1 × 10^−4^)	
Mutex [12]	0.730	0.650	ErbB Signaling Pathway (1.94 × 10^−9^) MAPK Signaling Pathway (2.8 × 10^−7^)	

Note: The genes in each row are sorted in descending order by their frequency of occurrence in the results.

**Table 3:** The identification of driver pathways in glioblastoma multiforme (GBM).

*k* (Number of Genes in Pathway)	The Driver Pathway
3	CDK4 CDKN2B RB1
4	CDK4 CDKN2B NF1 RB1
5	CDK4 CDKN2B EMP3 NF1 RB1
6	CDK4 CDKN2B EMP3 NF1 PRNP RB1
7	CDK4 CDKN2B NF1 FGFR TP53 RB1 CDKN2A
8	CDK4 CDKN2B NF1 FGFR TP53 RB1 CDKN2A SPHK2
9	FGFR NOTCH1 TP53 RB1 CDKN2A ZNF175 CDK4 CDKN2B NF1
10	CDK4 CDKN2B PTEN NF1 FGFR CDKN2A TP53 PRF1 RB1 SIGLEC9

Note: The genes in each row are sorted in descending order by their frequency of occurrence in the results.

**Table 4:** Comparison of the four methods for identifying pathways, submatrix weight, and accuracy for glioblastoma multiforme (GBM) data (**a**). Comparison of the four methods for coverage, mutual exclusion, and enrichment pathway for glioblastoma multiforme (GBM) data (**b**).

(**a**)				
**Method**	**Pathway**		**Submatrix Weight**	**Accuracy**
Our Method: MBF-Based	CDK4 CDKN2B PTEN NF1 FGFR CDKN2A TP53 PRF1 RB1 SIGLEC9		1.890	0.800
Dendrix [8]	CDKN2B ERBB2 SHH PI15 FGFR		1.540	0.600
MDPFinder [13]	CDK4 CDKN2B CSF1R ERBB2 FGFR3 HGF NTRK3 PRF1 RB1 SIGLEC9 CYP27B1 RB1 TP53 CDK4 CDKN2A EGFR CDKN2A TP53 MDM2		1.786	0.700
Mutex [12]	CDKN2B PTEN TRPV4 MYH1 RHOC HGF		1.393	0.5
(**b**)				
**Method**	**Coverage**	**Mutual** **Exclusion**	**Enrichment Pathway (q Value)**	
Our Method: MBF-Based	0.850	0.450	Glioma (1.83 × 10^−8^) Cell Cycle (2.73 × 10^−9^)	
Dendrix [8]	0.820	0.430	Pathways in Cancer (4.54 × 10^−11^) Bladder Cancer (1.98 × 10^−8^)	
MDPFinder [13]	0.760	0.370	Pathways in Cancer (1.12 × 10^−11^) Bladder Cancer (1.12 e× 10^−11^)	
Mutex [12]	0.730	0.380	Melanoma (9.34 × 10^−13^) P53 Signaling Pathway (5.74 × 10^−8^)	

Note: The genes in each row are sorted in descending order by their frequency of occurrence in the results.

## Data Availability

Not applicable.
